# Supplementary figures and images for: Pulsed electromagnetic field (PEMF) transiently stimulates the rate of mineralization in a 3-dimensional ring culture model of osteogenesis
Source: PLoS One. 2021 Feb 4;16(2):e0244223. doi: 10.1371/journal.pone.0244223 (PMC7861434; doi:10.1371/journal.pone.0244223)

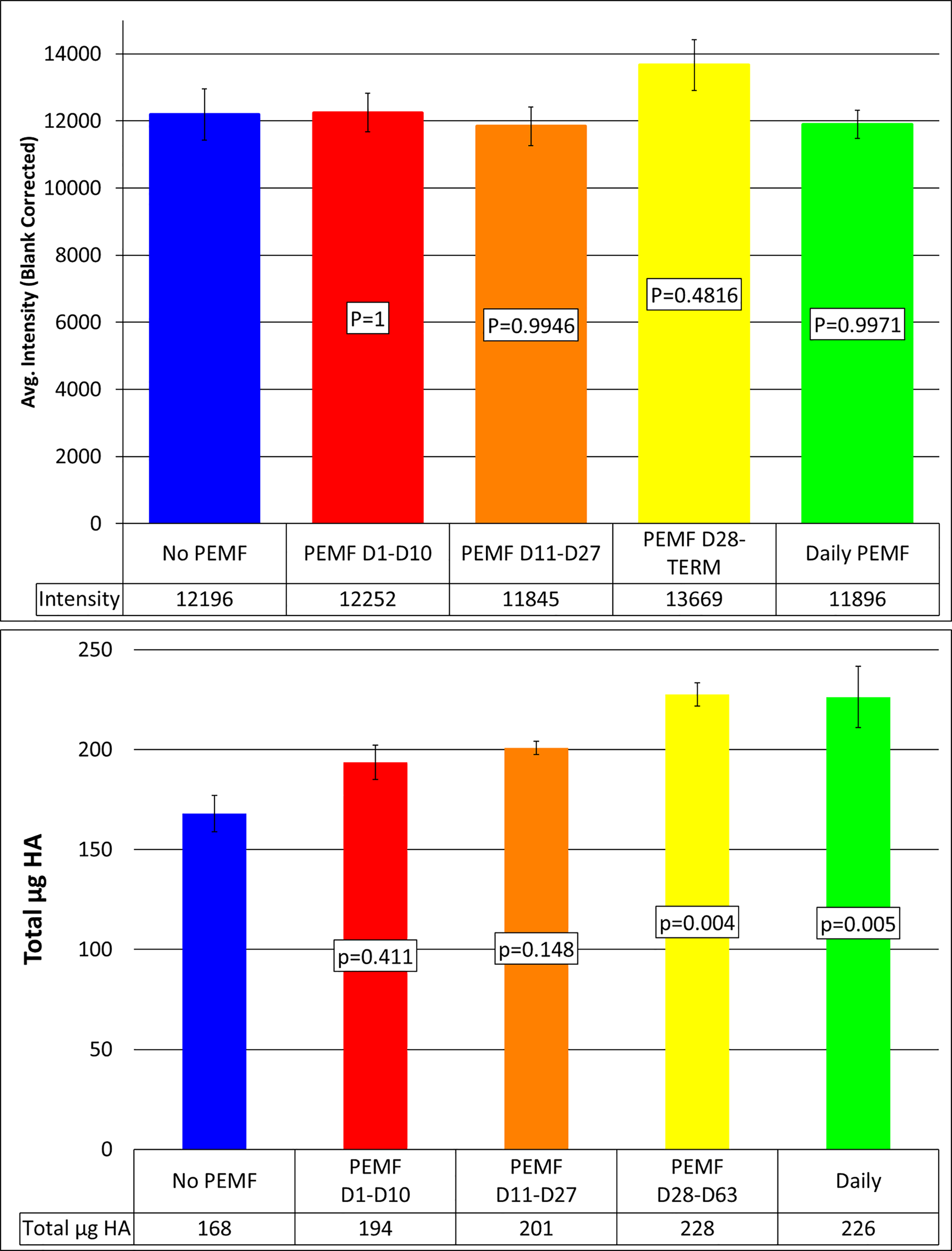

Supplement: S1 Fig — Mineralization measured by ARS (a) and by micro-CT (b) at Day 63. For ARS (a) P = Tukey HSD, groups not significantly different. For micro-CT (b), n = 4 per group, except Day 1-Day 11 where n = 3. Significant stimulation was detected for PEMF from Day 28-Day 63, and exposure to Daily PEMF. (ZIP) [file pone.0244223.s001.tif]

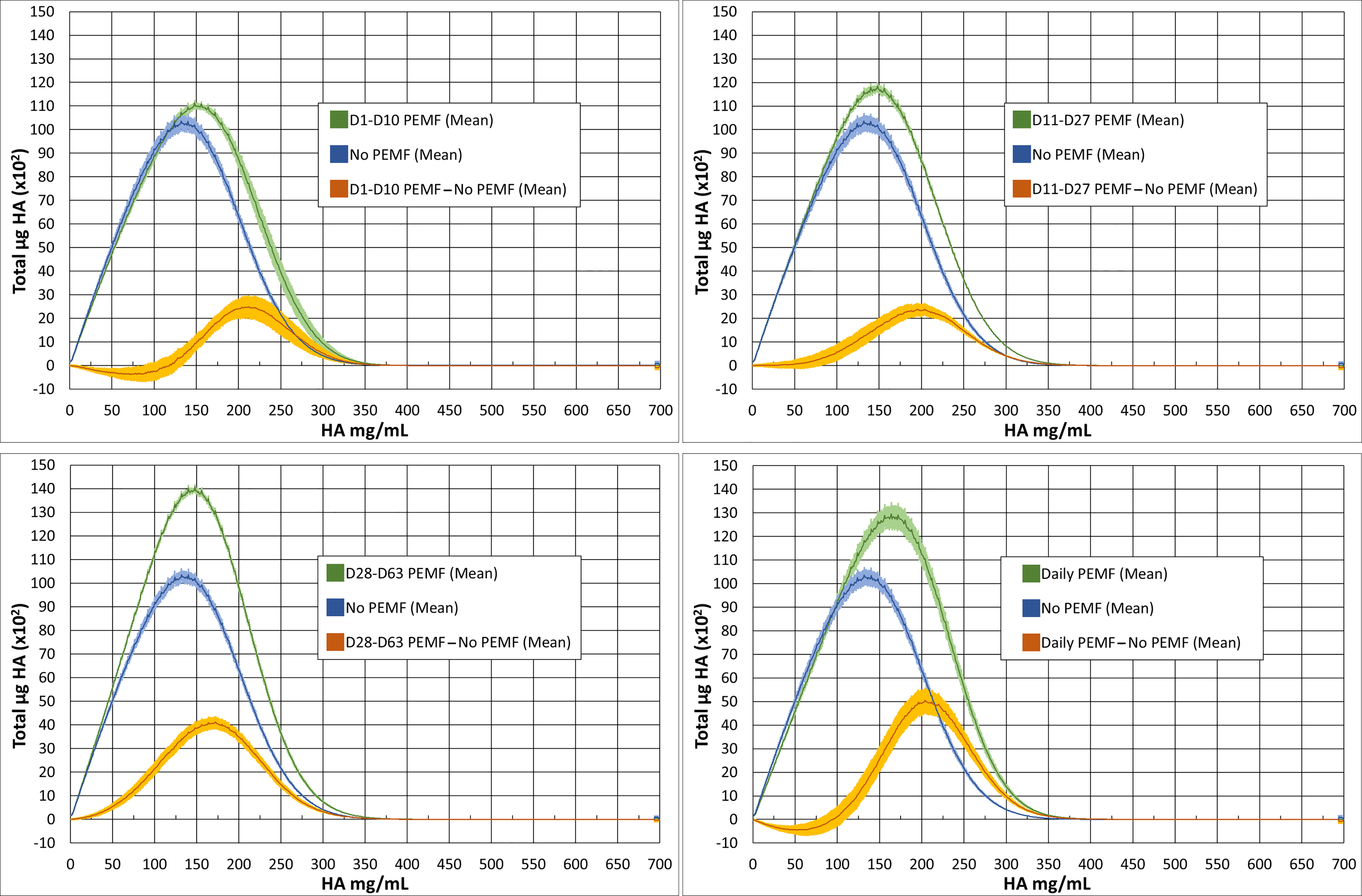

Supplement: S2 Fig — Total HA vs. HA/ml was plotted and profiles between no-PEMF controls and PEMF treatment between Day 1- Day 10 compared (a); PEMF between Day 11-Day 27 (b); PEMF between Day 28-Day 63 (c); and Daily PEMF (d). In each case n = 3, the line is the mean and its thickness represents the standard error. Control values were also subtracted from PEMF values at each density to yield a difference plot (orange) to visualize the effect of PEMF on mineral density. (ZIP) [file pone.0244223.s002.tif]

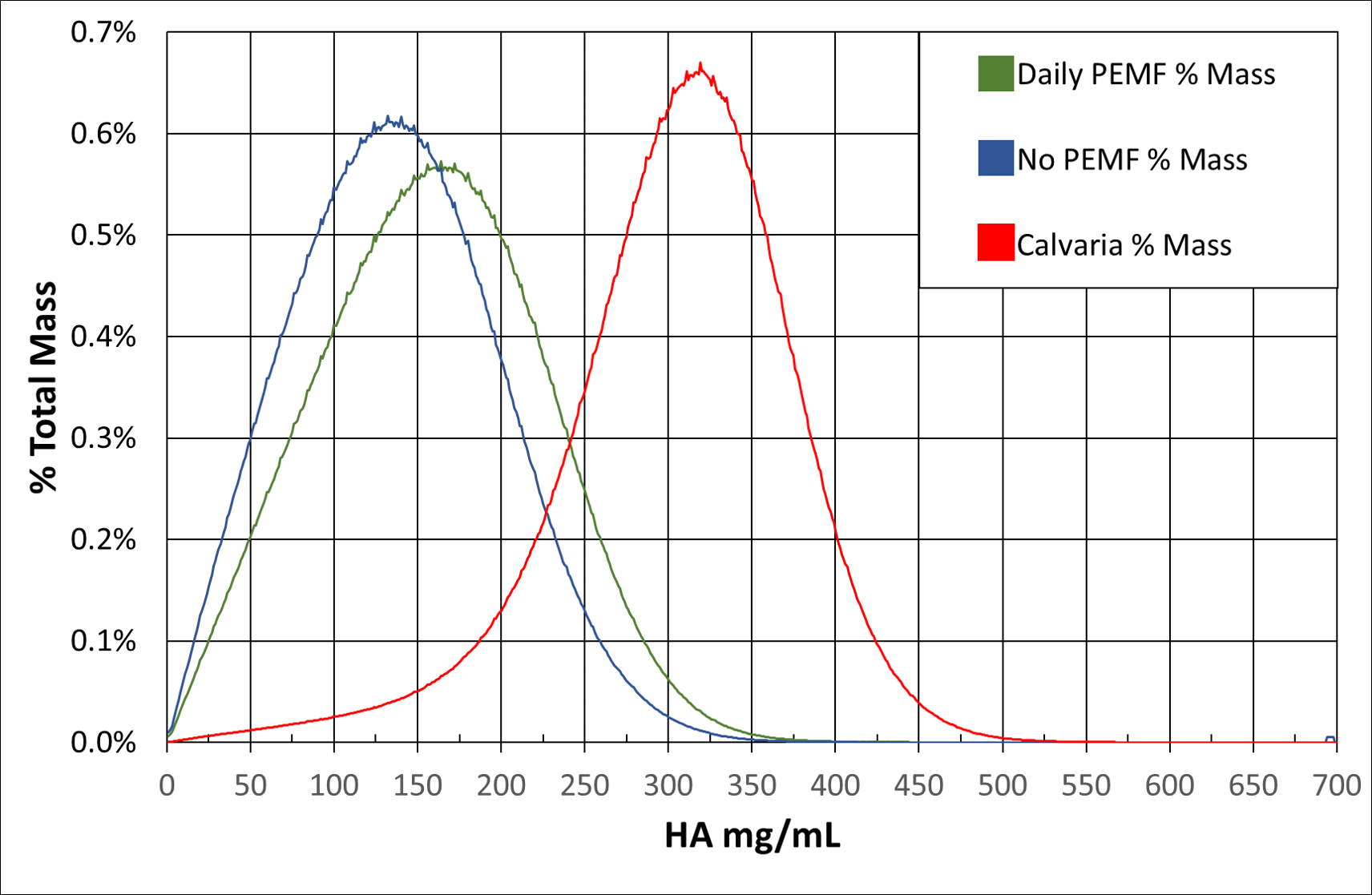

Supplement: S3 Fig — The mineral mass at each density was converted to a percentage of the total mass of the imaged construct/calvaria to allow easy comparison of the samples. For ring constructs n = 3, and the plotted line is the mean; for calvaria, n = 1. (TIF) [file pone.0244223.s003.tif]

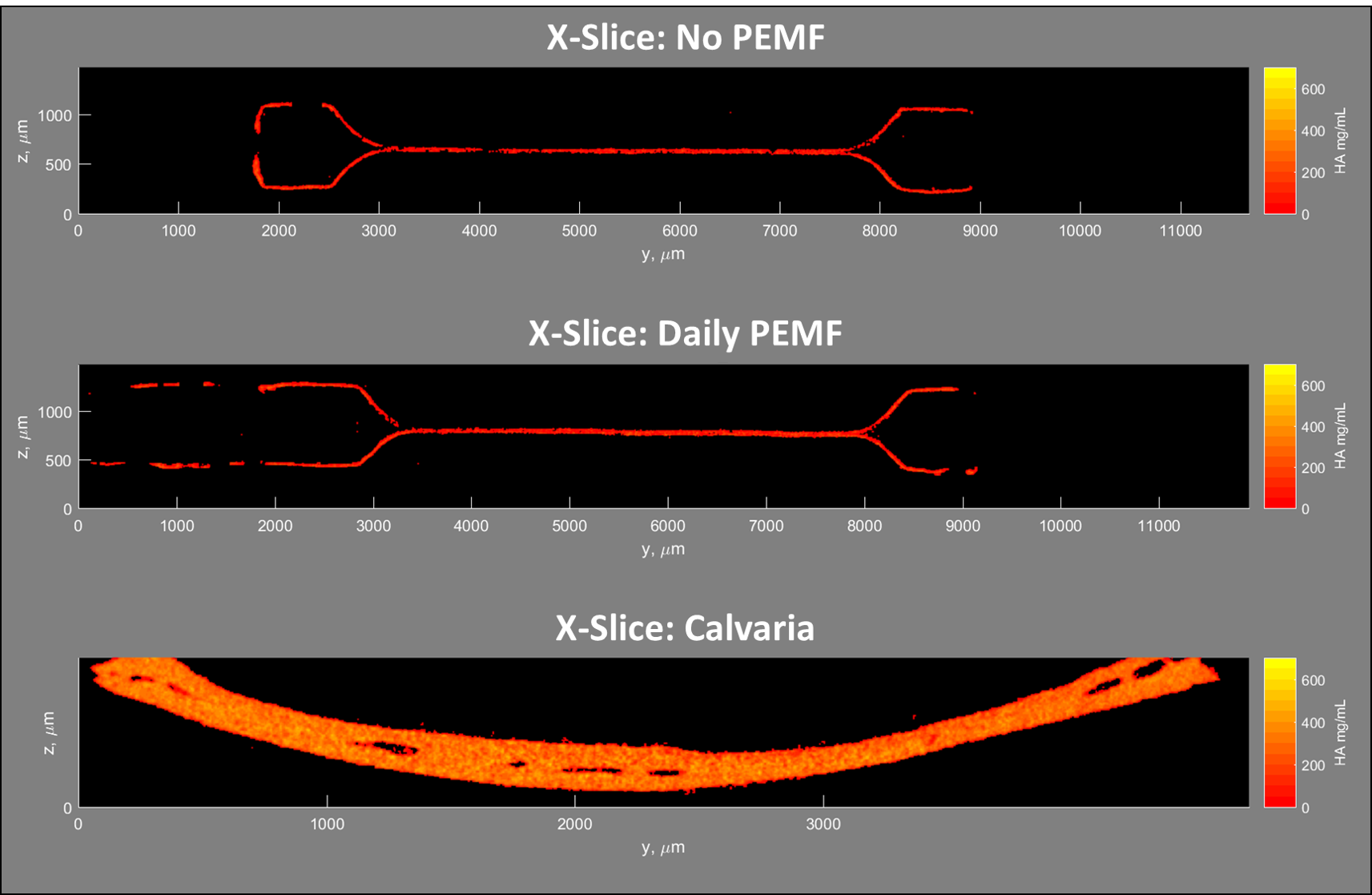

Supplement: S4 Fig — Computed cross-sections of micro-CT images on Day 63 of no-PEMF control constructs (a), Daily PEMF constructs (b), and 6-month-old calvaria (c). Sections were 10 microns thick. (TIF) [file pone.0244223.s004.tif]

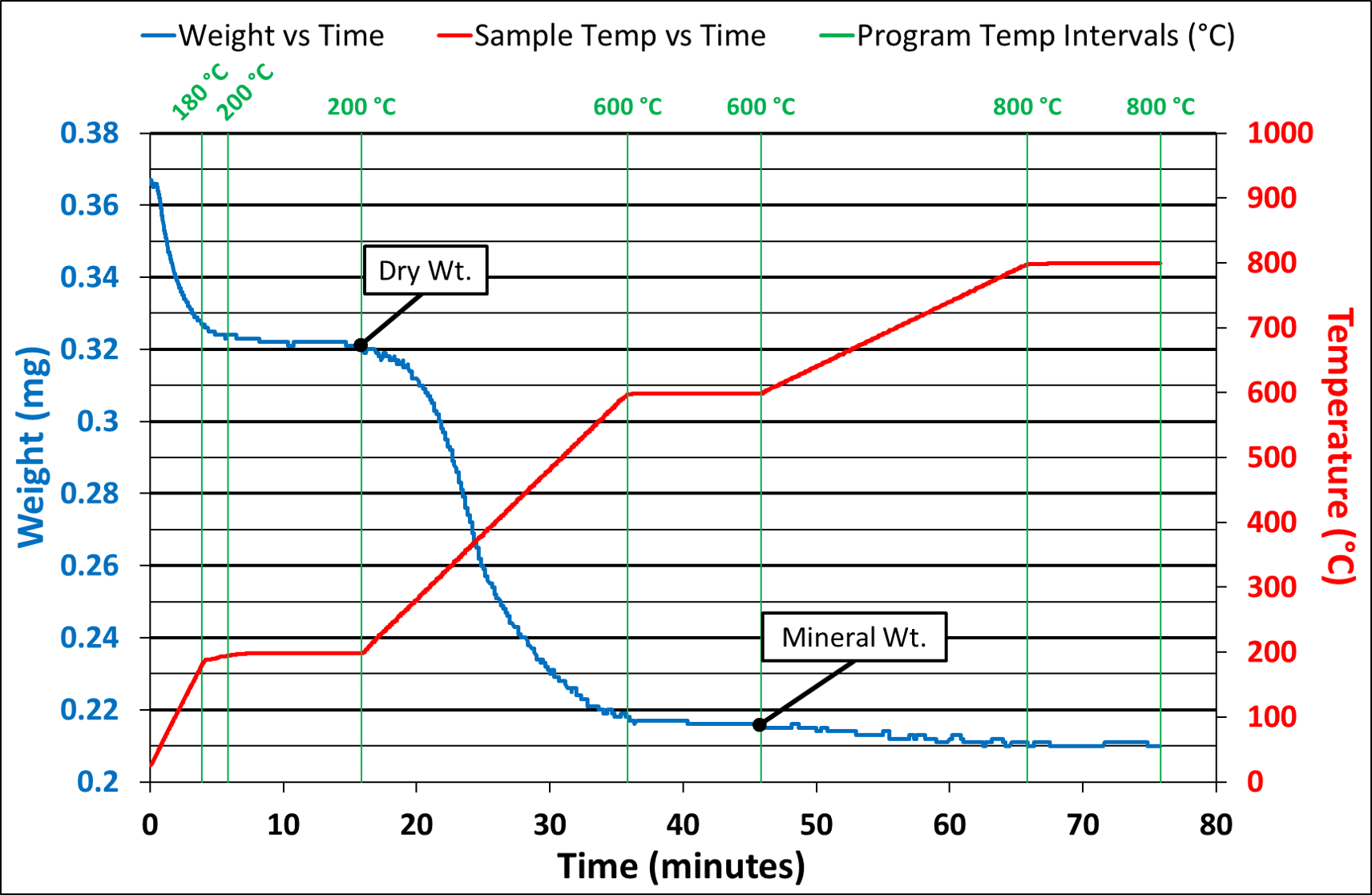

Supplement: S5 Fig — (TIF) [file pone.0244223.s005.tif]

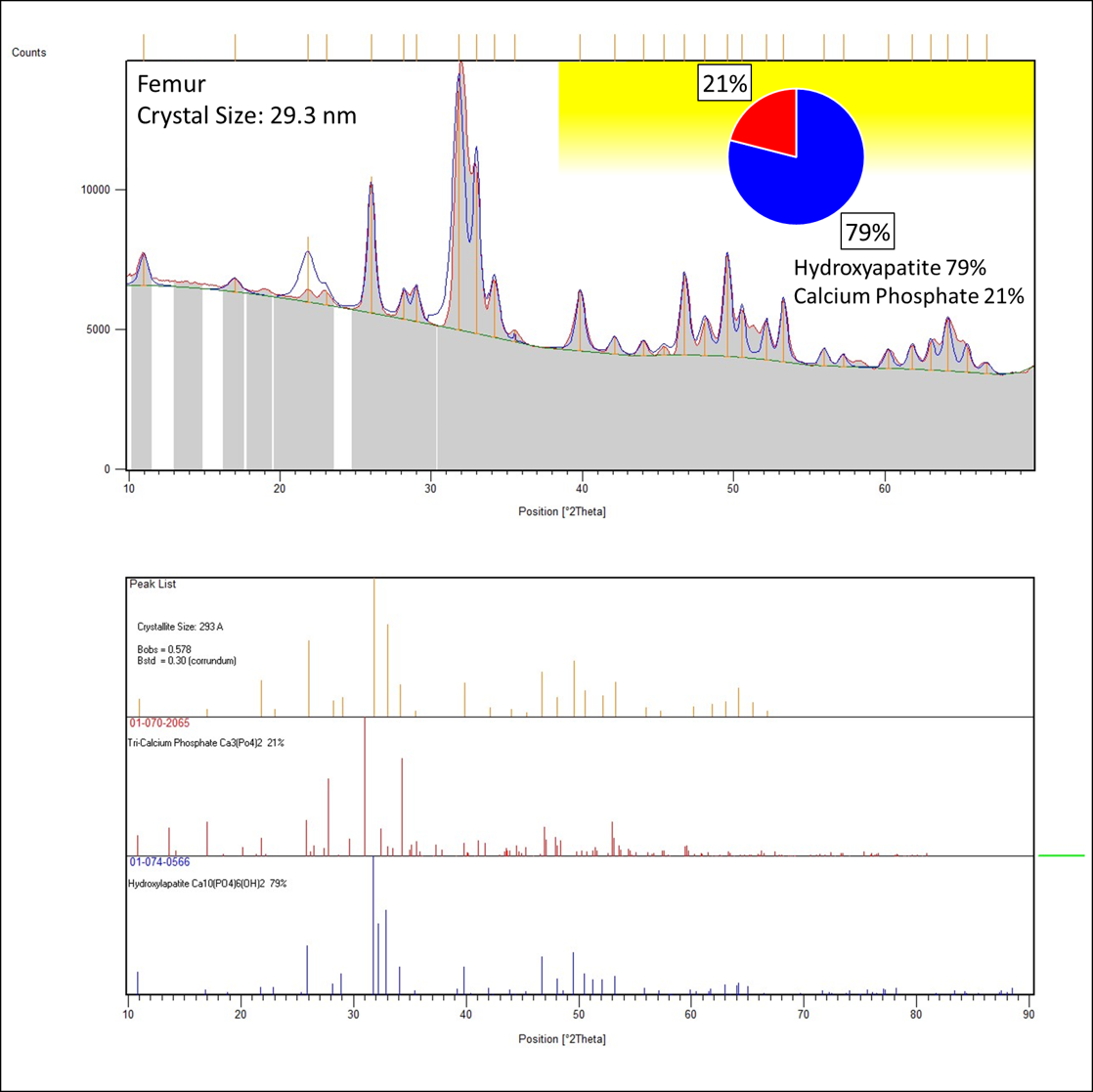

Supplement: S6 Fig — Mean crystallite size is in the top left corner. (TIF) [file pone.0244223.s006.tif]

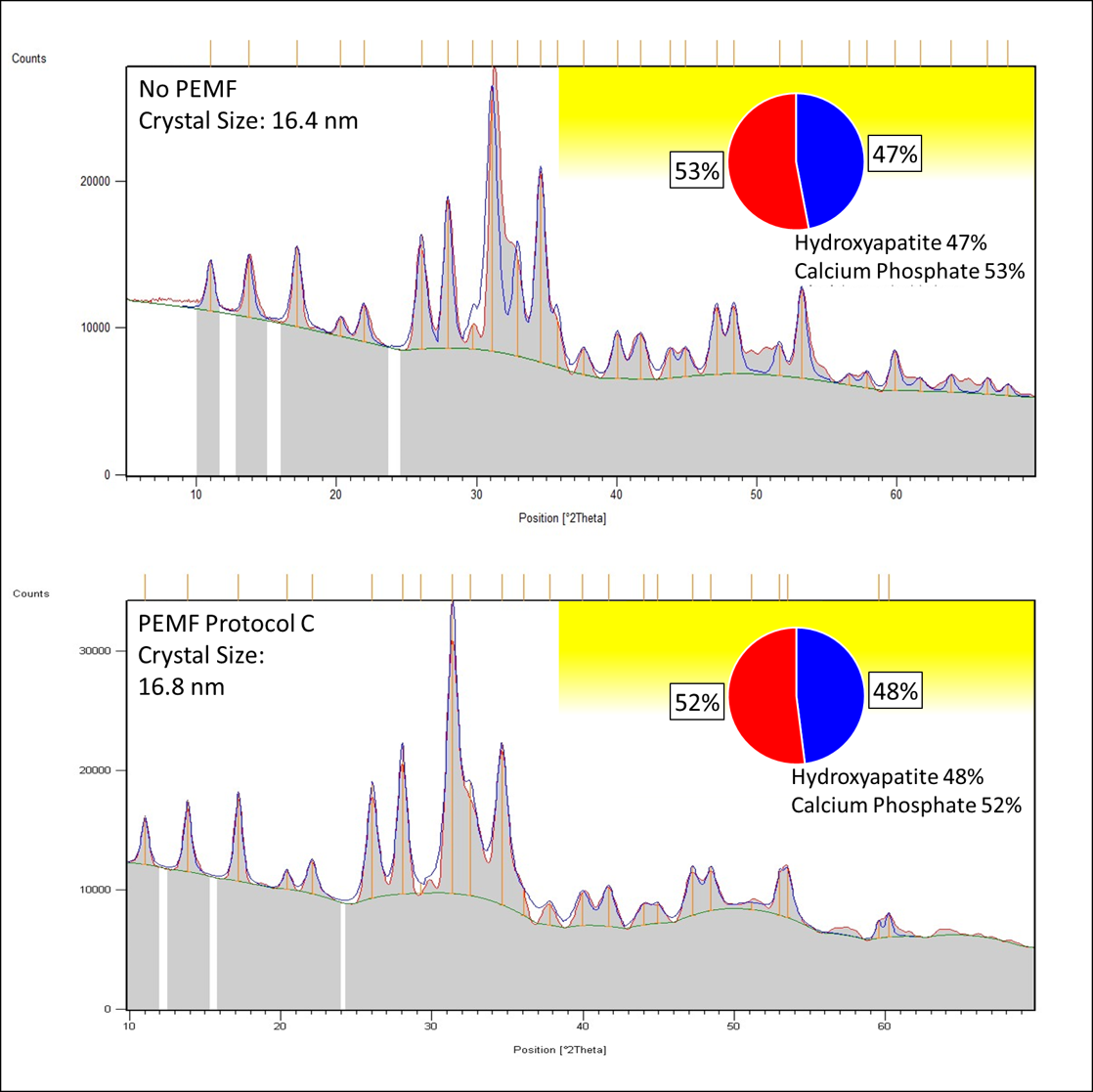

Supplement: S7 Fig — Mean crystallite size is in the top left corner. (TIF) [file pone.0244223.s007.tif]

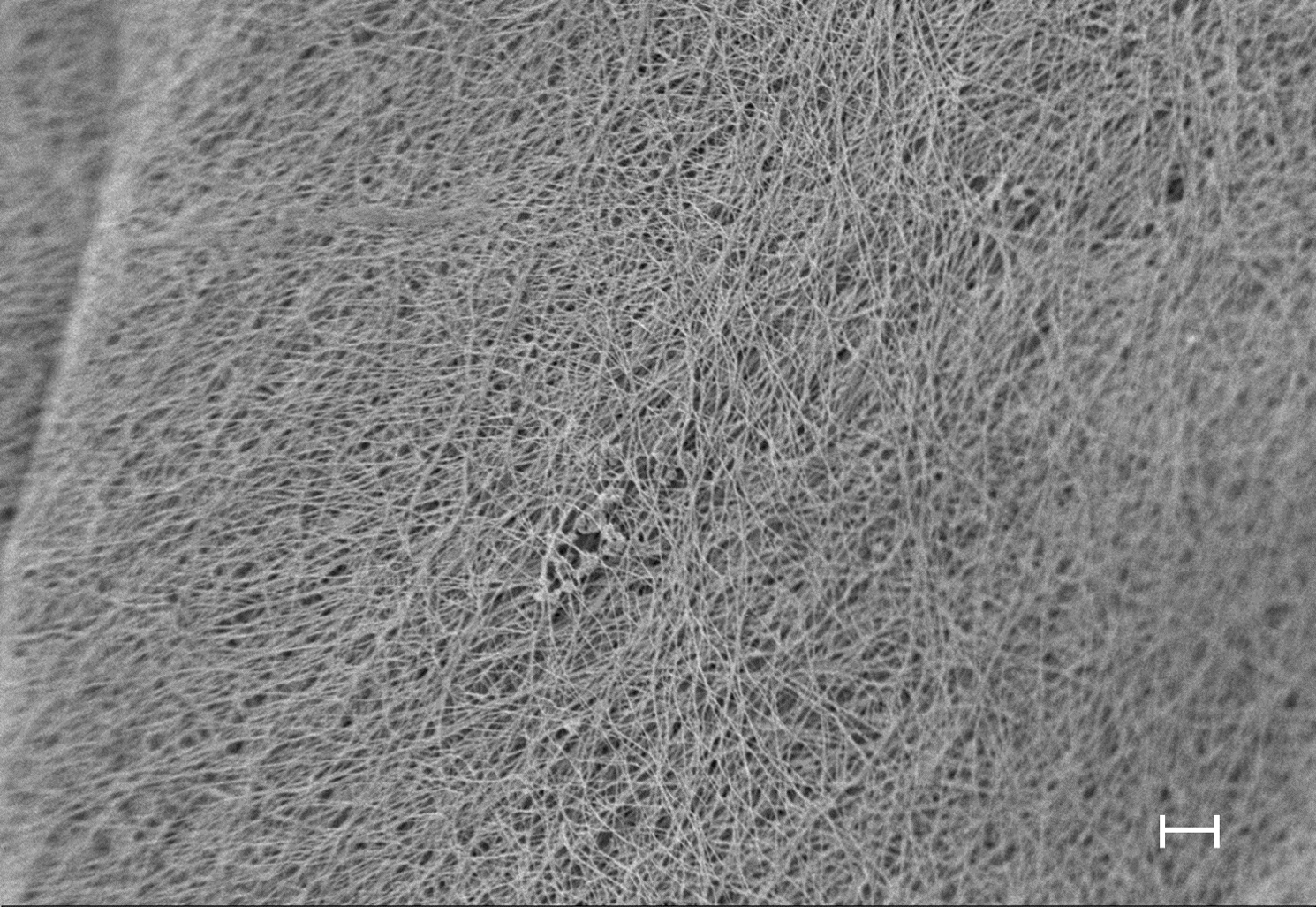

Supplement: S8 Fig — A fine mesh of collagen fibrils without large fibers contrasted with the deposited collagen architecture of the construct interior (Fig 2A). SEM settings: EHT = 1 KeV, WD = 2.4 mm, H = 17.42 μm, W = 23.23 μm, Mag = 4.92 K X. Scale Bar = 1 μm. (TIF) [file pone.0244223.s008.tif]

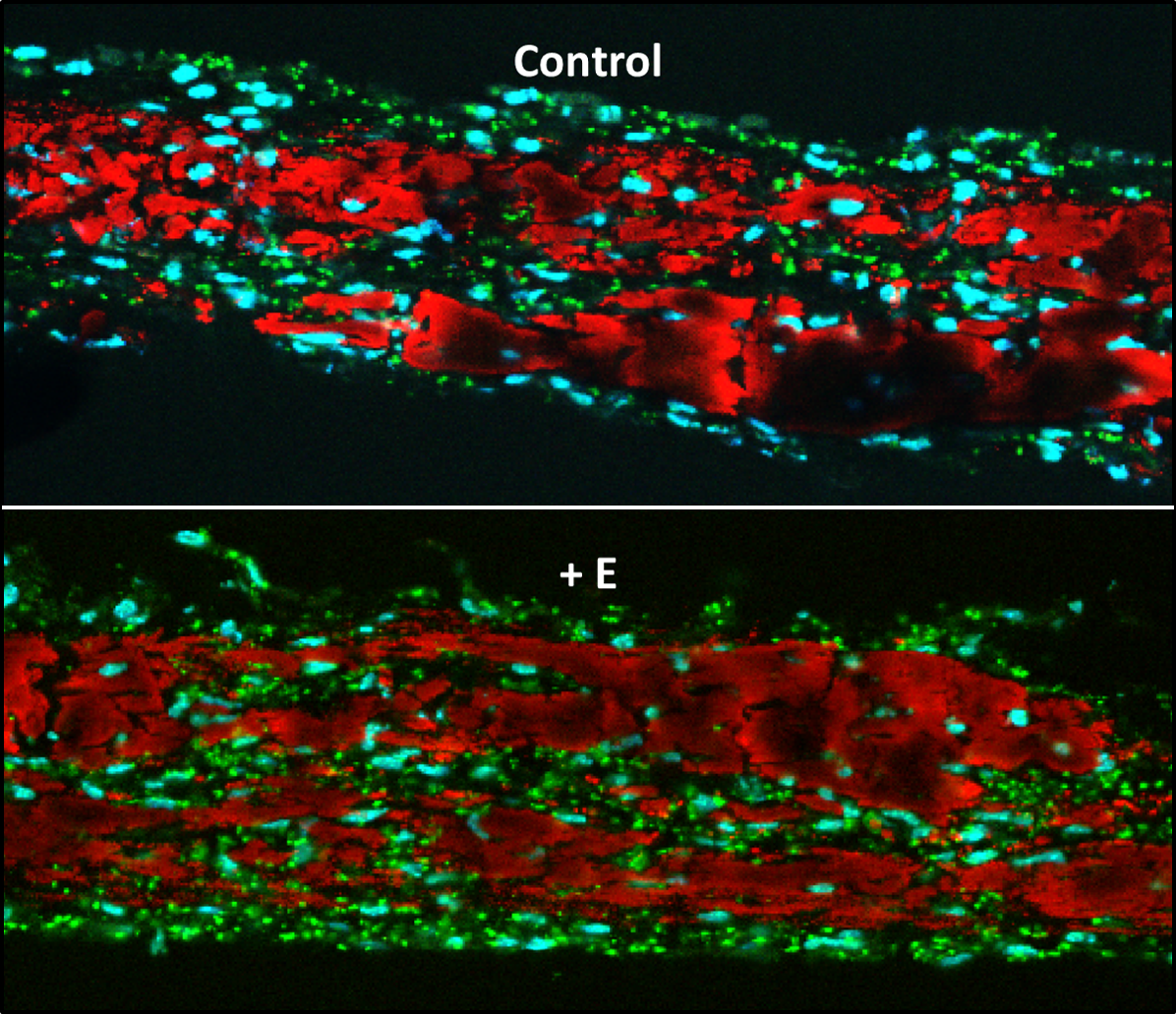

Supplement: S9 Fig — Note the increased continuity of mineral deposition and increased expression of alkaline phosphatase activity as a marker of the osteoblast lineage in the presence of estradiol, present in both Differentiation and Mineralization Media. (TIF) [file pone.0244223.s009.tif]

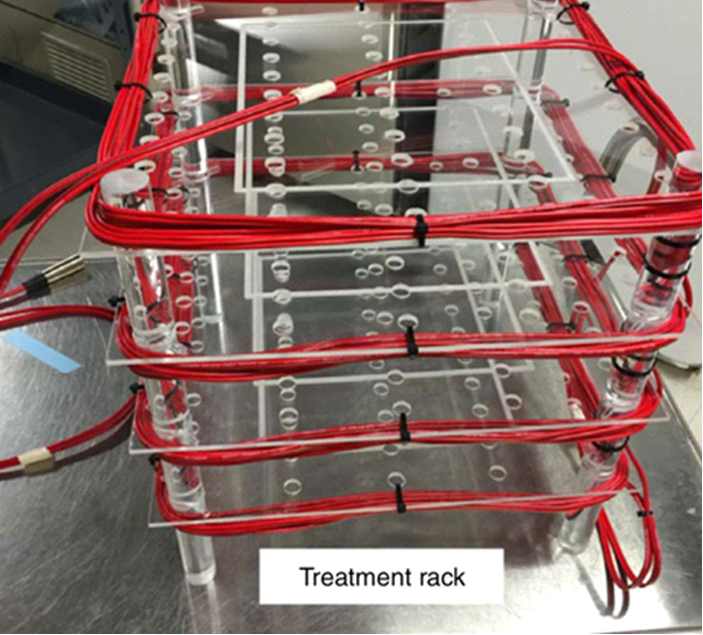

Supplement: S10 Fig — Note the rectangular culture treatment areas marked by white borders on each shelf. The center two shelves were routinely used. (TIF) [file pone.0244223.s010.tif]
